# Contribution of Fine Roots to Soil Organic Carbon Accumulation in Different Desert Communities in the Sangong River Basin

**DOI:** 10.3390/ijerph191710936

**Published:** 2022-09-01

**Authors:** Sihui Tian, Xin Liu, Baocheng Jin, Xuechun Zhao

**Affiliations:** 1 College of Animal Science, Guizhou University, Guiyang 550025, China; 2 State Key Laboratory of Vegetation and Environmental Change, Institute of Botany, Chinese Academy of Sciences, Beijing 100093, China

**Keywords:** desert communities, fine root, soil organic carbon, soil factors

## Abstract

This study explored the relationship between soil organic carbon (SOC) and root distribution, with the aim of evaluating the carbon stocks and sequestration potential under five plant communities (*Alhagi sparsifolia*, *Tamarix ramosissima*, *Reaumuria soongorica*, *Haloxylon ammodendron*, and *Phragmites communis*) in an arid region, the Sangong River watershed desert ecosystem. Root biomass, ecological factors, and SOC in different layers of a 0–100 cm soil profile were investigated. The results demonstrated that almost all living fine root biomass (11.78–34.41 g/m^2^) and dead fine root biomass (5.64–15.45 g/m^2^) levels were highest in the 10–20 cm layer, except for the *P. communis* community, which showed the highest living and dead fine root biomass at a depth of 60–70 cm. Fine root biomass showed strong seasonal dynamics in the five communities from June to October. The biomass levels of the *A. sparsifolia* (138.31 g/m^2^) and *H. ammodendron* (229.73 g/m^2^) communities were highest in August, whereas those of the *T. ramosissima* (87.76 g/m^2^), *R. soongorica* (66.29 g/m^2^), and *P. communis* (148.31 g/m^2^) communities were highest in September. The SOC of the five communities displayed strong changes with increasing soil depth. The mean SOC value across all five communities was 77.36% at 0–30 cm. The highest SOC values of the *A. sparsifolia* (3.08 g/kg), *T. ramosissima* (2.35 g/kg), and *R. soongorica* (2.34 g/kg) communities were found in June, and the highest value of the *H. ammodendron* (2.25 and 2.31 g/kg, *p* > 0.05) community was found in June and September. The highest SOC values of the *P. communis* (1.88 g/kg) community were found in July. Fine root production and turnover rate were 50.67–486.92 g/m^2^/year and 1.25–1.98 times per year. The relationships among SOC, fine root biomass, and ecological factors (soil water content and soil bulk density) were significant for all five communities. Based on the results, higher soil water content and soil conductivity favored the decomposition of root litter and increased fine root turnover, thereby facilitating SOC formation. Higher pH and bulk density levels are not conducive to soil biological activity and SOC mineralization, leading to increased SOC levels in desert regions.

## 1. Introduction

The soil carbon pool is one of the key carbon pools in terrestrial ecosystems and plays an essential role in the global carbon cycle [1,2]. Arid and semi-arid areas account for more than 40% of the terrestrial surface of Earth [3,4] and contain nearly 10% of the global soil organic carbon (SOC) stock [5]. Arid and semi-arid areas are extremely fragile ecosystems and are particularly sensitive to global change in the terrestrial ecosystems, and are characterized by their vulnerability to external environmental influences such as extreme weather, erratic precipitation, frequent droughts, and anthropogenic destruction [4,6]. Consequently, slight changes can have doubly significant impacts on the regional or global carbon cycle [7]. Although arid and semi-arid regions are important sources and sinks of carbon, long-term studies of SOC have focused on forests, grasslands, and croplands in humid and sub-humid regions [8,9], with forest soils storing more than 40% of total organic C in terrestrial ecosystems [10], grassland ecosystems storing more than 30% of SOC stock [11], and croplands storing almost 10% of the total global SOC reservoir in soils between 0–30 cm [12]. It is believed that these regions are more effective at carbon sequestration. However, arid and semi-arid regions not only absorb a large amount of CO_2_ through the neutralization of saline-alkali soil, in which the CO_2_ is stored in the soil as inorganic carbon [13], but a considerable amount of CO_2_ is fixed via vegetation uptake and turned into organic carbon, which is more stable [14]. The absorption rate of CO_2_ by saline-alkali soil is 62–622 g C/m^2^/a [15], and the amount of C absorbed by bio-communities in temperate deserts is equivalent to that of temperate forests and grassland ecosystems.

In arid and semi-arid ecosystems and other terrestrial ecosystems, the difference between inputs to primary production and the carbon returned to the atmosphere through organic matter decomposition determines the soil carbon budget [16]. Plant communities, the main source of soil organic carbon in terrestrial ecosystems, can input organic carbon sequestered by photosynthesis into the soil through litter decomposition and root life activities (e.g., fine root turnover). It is estimated that 22–60% of the carbon sequestered by plant photosynthesis [17,18] is allocated to the belowground root system to sustain the continued growth, death, and renewal of fine roots, with several times more organic matter entering the soil through the fine root death and turnover pathway than through the decomposition of aboveground litter [19,20,21]. Although fine roots account for a tiny proportion (<5%) of the total standing root biomass [16], the growth, respiration, and turnover of fine roots are predicted to absorb more than 33% of worldwide net primary output in diverse terrestrial ecosystems [17,18,22,23]. Notably, compared with species in wet and sub-humid habitats, communities of desert ecosystems generally invest more carbon for the development of their root system and form complex root networks due to environmental stress [18,24,25]. Moreover, root tissues remain in the soil longer than other plant tissues [21,25,26]; therefore, there may be considerable C transfer from vegetation to the soil through root turnover [27]. Especially in resource-limited desert ecosystems, the fine root distribution and biomass of vegetation communities strongly influence the SOC depth distribution both for accessing basic resources and for building vegetation patterns [28,29]. In this sense, it is critical to observe the dynamic changes in fine root biomass and estimate fine root production and turnover to reliably assess the SOC budget in different desert communities.

The unusually high spatial heterogeneity of soil resources is an intrinsic characteristic of desert ecosystems, resulting in fine-root dynamics in desert areas being more susceptible to more complex interactions of environmental and biological factors [30]. Among the environmental factors, precipitation and temperature play a dominant role in plant growth and biomass allocation, while in arid regions, the main controlling factor for fine root decomposition is temperature [2]. In addition, soil water content, soil bulk density, pH, and soil electrical conductivity also influence fine root dynamics, turnover, and decomposition, which, in turn, affect SOC income and expenditure [31].

The determination of plant belowground root biomass is a prerequisite for the study of fine root production and turnover, and is the basis for the study of the process of fine root turnover to soil carbon input. In this study, the sequential soil coring method was used, which causes little disturbance to the soil environment of the root samples and is currently the most commonly used estimation method [32]. The production and turnover of plant fine roots were calculated by collecting soil cores at successive intervals, and by separating and identifying the live and dead fine roots in the soil. Several studies have been conducted on the aboveground parts of some desert plant communities, such as studies on the morphological characteristics, community characteristics, and stress resistance, as well as on the input of organic carbon to soils in saline areas by litter and root turnover [27,31,33] In this study, the plant communities (*Alhagi sparsifolia*, *Tamarix ramosissima*, *Reaumuria soongorica*, *Haloxylon ammodendron*, and *Phragmites communis*) were the dominant species in the desert area. Although the contribution of fine roots to SOC is greater in desert plant communities, some of the mechanisms remain unclear. Therefore, we investigated the biomass and turnover of fine roots in desert communities, explored the contribution of fine roots to the vertical distribution of SOC in deeper soil depths (0–100 cm), and analyzed the effect of soil factors on SOC in five plant communities in depth. This study aimed to evaluate the contribution of fine root biomass to carbon stocks and sequestration potential in desert plant communities and to provide a theoretical basis and data support for research on soil carbon in arid and semi-arid ecosystems.

## 2. Materials and Methods

### 2.1. Study Area

**Figure 1:**
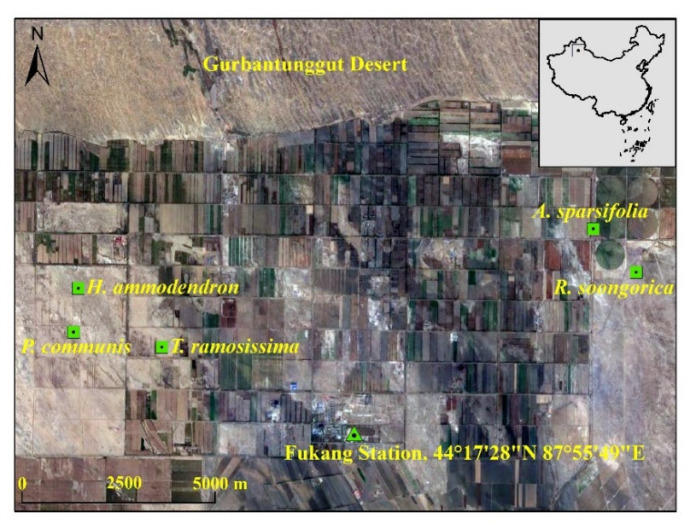
Location of the study area and experimental sites (*Haloxlon ammodendron*, *Phragmites communis*, *Tamarix ramosissima*, *Alhagi sparsifolia*, and *Reamuria soongorica* communities).

The present research was conducted at the Fukang Station of Desert Ecology, Xinjiang Institute of Ecology and Geography, Chinese Academy of Sciences (43°45′–45°29′ N 87°43′–88°44′ E), at the southern edge of the Junggar Basin (Figure 1). The region has a typical temperate continental arid climate, with a mean annual rainfall of 160 mm and a mean annual temperature of 6.6 °C. The natural vegetation is dominated by *Reaumuria soongorica* (Pall.) Maxim, *Haloxylon ammodendron* (C. A. Meyer) Bunge, *Tamarix ramosissima* Ledeb, *Alhagi sparsifolia* Shap, and *Phragmites communis* (Cav.) Trin. ex Steud. The soil type is gray desert salt-alkali soil and dry sandy loam soil. Five plant communities were selected for analysis, based on the dominant natural vegetation. Three randomly selected 25 × 25 m experimental plots were established in each of the five communities, resulting in a total of 15 plots (Table 1).

### 2.2. Fine Root Biomass, Production, and Turnover Rate

Each of the five communities was used to investigate the seasonality of live fine root biomass and turnover rate in each month from May to October 2010. Fine root production from May to October was used to represent the annual fine root production. We considered the average distance (D) between two dominant plants as the standard, and samples were collected in three quadrats, i.e., near the plant, at 1/4 D, and at 1/2 D. Each fine root sample was taken from three plots of the freshly cut profile walls by collecting a 50 × 50 cm soil core. The soil core samples were divided into 10 soil depths of 0–10, 10–20, 20–30, 30–40, 40–50 and 50–60, 60–70, 70–80, 80–90, and 90–100 cm until few fine roots were found. The collected fine root samples were sealed in plastic bags and transported to the laboratory. In the laboratory, roots with a diameter more than 2 mm were discarded, and live and dead fine roots less than 2 mm were manually washed and separated based on their color and luster, resilience, consistency, smell, degree of cohesion between the cortex and the periderm, and the appearance of the phloem. The separated fine roots were stored on the basis depth interval and oven-dried to a constant mass at 65 °C for determination of the mass and for chemical analysis.

Fine root production and turnover were estimated and calculated on the basis of the maximum–minimum method, specifically, based on the following equations: [20,34]:

(1) M=Mmax – Mmin +D

(2) P=Pmax – Pmin +M

(3) T=P/Pa

where *M*, *D*, *P*, and *T* indicate the annual fine root mortality, decomposition, production, and turnover, respectively. *Mmax* and *Mmin* are the maximum and minimum values of dead fine root biomass, whereas *Pmax*, *Pmin*, and *Pa* are the maximum, minimum, and average values of live fine root biomass during the entire growing season.

### 2.3. Measurement of Fine Root Decomposition

A decomposition experiment with fine roots was established in each of the five communities in 2010, which were collected from the top 100 cm of the soil in each of the five communities by sieving roots during the 2009 growth season. Nylon mesh bags (15 × 20 cm and 0.12 mm mesh size) were used for determining root decomposition [35]. Approximately 5 g of fine-root samples and 500 g of the soil in which the fine roots had been sampled were placed in the five ecosystems at about a depth of 20–30 cm in the soil horizon in early May 2010. Bags were deployed randomly in the five ecosystems using the coordinates of permanently marked grids. Three or more bags from each ecosystem were collected once a month. Upon collection, bags were immediately returned to the laboratory, the fine soil particles were removed, and fine roots were removed from bags, dried at 65 °C to a constant mass, and weighed. The fine root samples were ground and passed through a 100-mesh sieve for subsequent analyses.

The fine root exponential decay function (4) was used to fit the curve of the percentage of mass remaining, and the time of decomposition incubation and annual fine root decomposition were calculated according to the fitted functions [36], using the following equation:

(4) $$W_{t}=W_{0}e^{-kt}$$

where $W_{0}$ is the initial dry weight, $W_{t}$ is the dry weight remaining after the decomposition interval, *k* is the decomposition coefficient, and *t* is the decomposition interval (days).

### 2.4. Soil Physical and Chemical Properties

Soil samples were collected in similar places to the fine root samples at a depth of 100 cm at seven intervals of 0–5, 5–10, 10–20, 20–30, 30–40, 40–50 and 50–100 cm with a cutting ring. The soil samples were naturally dried and sieved through a 2 mm mesh size in order to remove plant materials, small insects, and stones. Soil pH (1:5 solid-to-water (*w*/*v*)) and soil electrical conductivity (SEC) (1:5 solid-to-water (*w*/*v*)) were determined in deionized water with a Eutech PC700 pH/EC meter (Thermo Fisher Scientific Inc., Waltham, MA, USA). Soil bulk density (SBD) was determined for each depth interval using a cutting ring and calculated as the ratio of oven-dry soil weight to cutting ring volume. Soil water content was determined by drying for 3 days at 105 °C. The rest of the soil samples were air-dried and ground to pass through a 100-mesh sieve for subsequent analyses.

Soil bulk density (*SBD*, g/cm^3^) and soil water content (*SWC*, %) were calculated using Equations (5) and (6), respectively:

(5) $$SBD=\;M_{S}/V_{t}$$

(6) $$SWC=(M_{W}\;-M_{S})/M_{W}$$

where $M_{S}$ is the dry soil weight (g), $V_{t}$ is the total volume of the soil core (cm^3^), and $M_{W}$ is the fresh soil weight (g).

### 2.5. Measurement of SOC and Fine Root Organic Carbon

Soil carbon and fine root carbon were determined by a wet oxidation technique [37]. The fine root carbon input was determined using Equation (7):

(7) $$C_{input}=D\;\times \;FOC$$

where *C_input_* is the fine root organic carbon input to soil by decomposition (g/m^2^/a) and *FOC* is the fine root organic carbon content (%).

An asymptotic nonlinear model was used to describe the SOC stock’s depth distribution, using the following Equation (8) [18,38,39]:

(8) $$y\;={\;1\;–\;\beta }^{\;d}$$

where y is the cumulative SOC content from the soil surface to a certain soil depth *d* in centimeters, and the *β* value describes the shape of the vertical distribution of SOC within the whole sampled soil profile. High values of *β* correspond to a larger proportion of SOC at greater soil depths.

### 2.6. Data and Statistical Analysis

All calculations were performed using SPSS 16.0 software (SPSS Inc., Chicago, IL, USA), including ANOVA and homogeneity of variance tests, multiple comparisons, and regression analyses. The differences in the fine root productivity, decomposition, decomposition of supplementary soil organic carbon (SOC), and turnover rate of the five plant communities were compared by the LSD test. The dependence of SOC on all variables and soil depths were analyzed further by regression analysis. Mean differences were considered significant at *p* < 0.05. SigmaPlot for Windows Version 10.0 was used for graphics.

## 3. Results

### 3.1. Fine Root Vertical Distribution and Seasonal Dynamics

**Figure 2:**
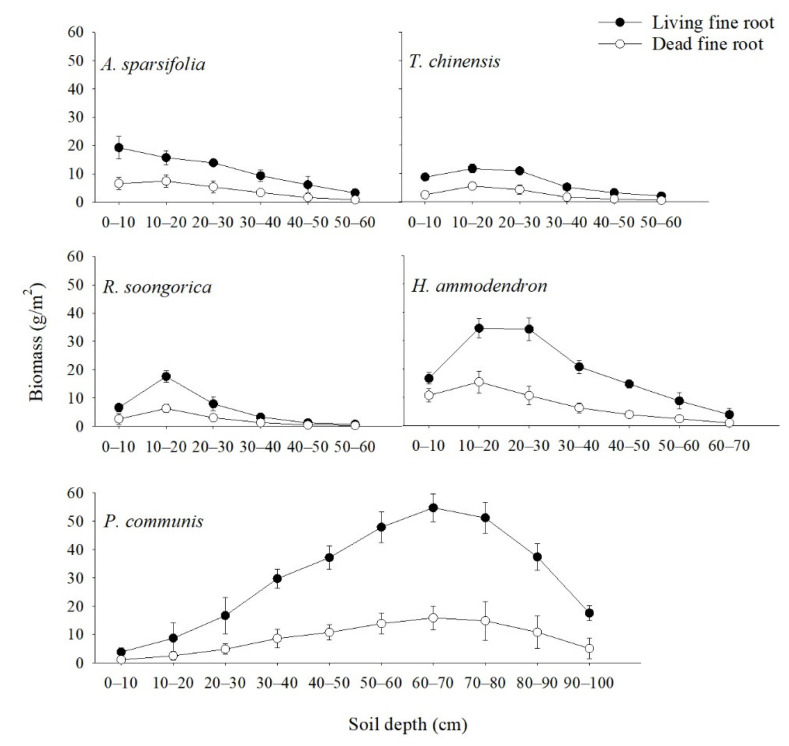
Living and dead fine root biomass (mean ± SD) of the five plant communities at different soil depths. Each value represents the average of three replicates.

The vertical distribution of living fine root biomass first increased and then decreased with soil depth in the five communities. However, in the *A. sparsifolia* community, it only decreased with soil depth. Living fine root biomass was highest in the 10–20 cm soil profile, except for the *A. sparsifolia* and *P. communis* communities (Figure 2). The highest living fine root biomass at 10–20 cm was 11.78–34.41 g/m^2^ for the *T. ramosissima*, *R. soongorica*, and *H. ammodendron* communities, whereas the highest living fine root biomass at 0–10 cm and 60–70 cm was 19.25 and 54.79 g/m^2^ for *A. sparsifolia* and *P. communis*, respectively. The fine root biomass was mainly concentrated at a depth of 0–30 cm. The living fine root biomass at a depth of 0–30 cm accounted for 63.86–86.0% of the total living fine root biomass for the *A. sparsifolia*, *T. ramosissima*, *R. soongorica*, and *H. ammodendron* communities, but living fine root biomass at 40–90 cm accounted for 84.65% of the total living fine root biomass for the *P. communis* community.

The vertical distribution of dead fine root biomass first increased and then decreased with soil depth. Dead fine root biomass was highest at 10–20 cm, except for the *P. communis* community (Figure 2). The highest dead fine root biomass at 10–20 cm was 5.64–15.45 g/m^2^ for the *A. sparsifolia*, *T. ramosissima*, *R. soongorica*, and *H. ammodendron* communities. The highest dead fine root biomass at 60–70 cm was 15.89 g/m^2^ for the *P. communis* community. The dead fine root biomass at 0–30 cm accounted for 73.04–85.03% of the total dead fine root biomass for the *A. sparsifolia*, *T. ramosissima*, *R. soongorica*, and *H. ammodendron* communities, and the dead fine root biomass at 40–90 cm accounted for 84.65% of the total dead fine root biomass for the *P. communis* community.

**Figure 3:**
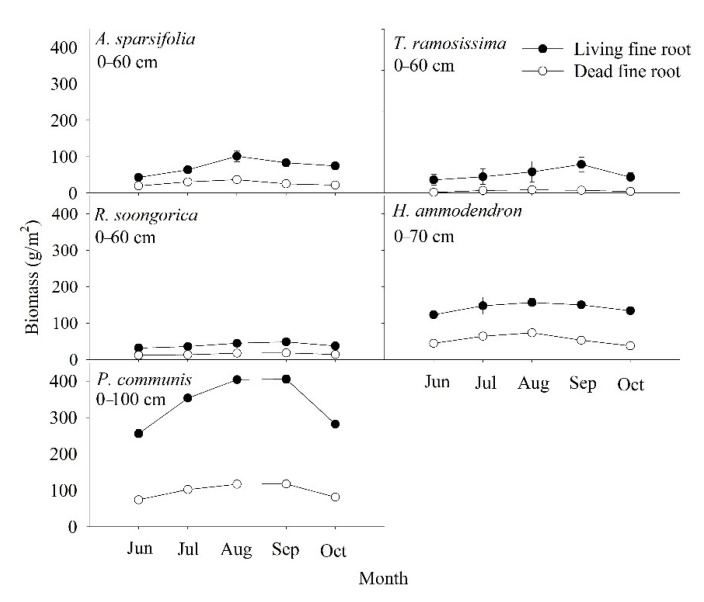
Seasonal living and dead fine root biomass (mean ± SD) of the five plant communities at different soil depths. Each value represents the average of three replicates.

From June to October, the living and dead fine root biomass of the five communities showed strong seasonal dynamics, increasing and then decreasing (Figure 3). Living fine roots in five communities peaked in August or September (48.45–405.75 g/m^2^), with the lowest values found in June (31.40–255.73 g/m^2^). The maximum value of living fine root biomass (405.75 g/m^2^) occurred in September for *P. communis*, and the minimum value of living fine root biomass (31.40 g/m^2^) occurred in June for *R. soongorica*. Dead fine root biomass in the five communities peaked in June, July, or August, with the maximum value of dead fine root biomass (23.48 g/m^2^) found in July for *H. ammodendron*; the minimum value was found in June, September, and October; and the minimum value for *T. ramosissima* (1.72 g/m^2^) found in June.

### 3.2. Fine Root Biomass and Production

**Figure 4:**
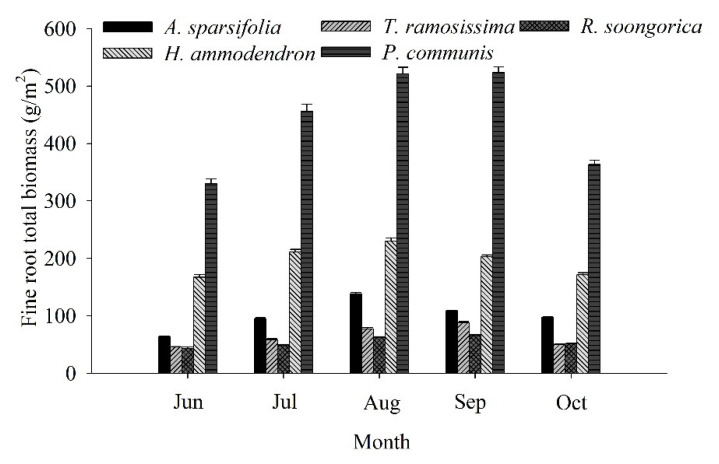
Seasonal fine root biomass (mean ± SD) at 0–100 cm of the five plant communities. Each month represents the average of three replicate plots.

Fine root biomass showed strong seasonal dynamics in the five communities from June to October (Figure 4). The highest fine root biomass was found for the *P. communis* community and the lowest was for the *R. soongorica* community. The monthly mean fine root biomass was 54.19–438.89 g/m^2^ for the five communities. The seasonal variation in biomass was similar between the *A. sparsifolia* and *H. ammodendron* communities, with an initial increase followed by a decrease; the highest values of 138.31 and 229.73 g/m^2^, respectively, were measured in August. The seasonal variation in biomass was similar among the *T. ramosissima*, *R. soongorica*, and *P. communis* communities; biomass first increased and then decreased, with the highest values of 66.29–523.41 g/m^2^ being measured in September.

### 3.3. SOC Vertical Distribution and Seasonal Variations

**Figure 5:**
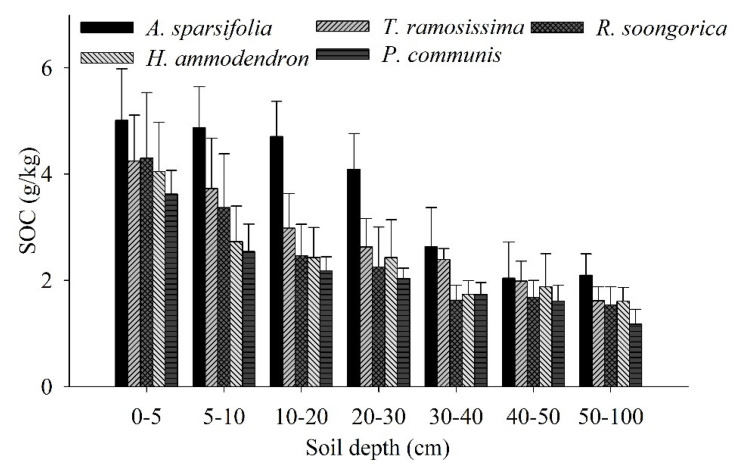
SOC (mean ± SD) of the five plant communities at soil depths of 0–100 cm. Each value represents the average of 3 three replicates.

The SOC of the five communities changed greatly with increasing soil depth (Figure 5). The *A. sparsifolia* community showed the highest SOC content in all soil layers compared with the other four communities. In addition to the 30–40 cm soil layer, the *P. communis* community in all soil layers had the lowest SOC content compared with the other four communities.

**Figure 6:**
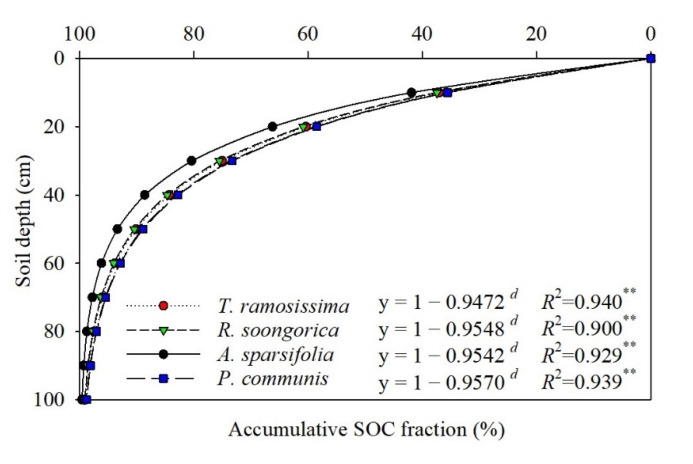
Asymptotic single−parameter global model of the vertical distribution of soil organic carbon (SOC) of the five plant communities in 0–100 cm. Note: *d*, soil depth (cm); Significance levels: **, *p* < 0.01.

The *β* values of the SOC ranged from 0.9472 to 0.9570 for the five communities (Figure 6). Among all communities, the accumulation of SOC with soil depth differed significantly (Figure 5). The mean SOC value was only 74.36% for the *H. ammodendron*, *T. ramosissima*, *R. soongorica*, and *P. communis* communities at 0–30 cm, whereas for the *A. sparsifolia* community, 80.36% of the SOC was distributed in the 0–30 cm soil layer.

The SOC contents in the five communities varied strongly with seasons (Table 2). The highest monthly mean values of SOC occurred in the *A. sparsifolia* community and the lowest ones in the *P. communis* community. The monthly average values were 1.65–2.89 g/kg for the five communities. The highest SOC values of the *A. sparsifolia* (3.08 g/kg), *T. ramosissima* (2.35 g/kg), and *R. soongorica* (2.34 g/kg) communities were found in June, and the highest values for the *H. ammodendron* (2.25 and 2.31 g/kg, *p* > 0.05) community were found in June and September, and the lowest ones were 2.79, 2.07, 1.68, and 1.62 g/kg, respectively. The lowest and highest SOC values of the *P. communis* communities were 1.19 g/kg in June and 1.88 g/kg in July.

### 3.4. Effects of Fine Roots on SOC Dynamics

Fine root production, annual decomposition, and SOC supplementation followed the order *P. communis* > *H. ammodendron* > *A. sparsifolia* > *T. ramosissima* > *R. soongorica*. The fine root productivity of the *P. communis* community was 9.61 times that of the *R. soongorica* community. In this study, fine root production was 50.67–486.92 g/m^2^/yr, decomposition was 16.08–130.24 g/m^2^/yr, and supplementary SOC was 7.14–50.42 g/m^2^/yr. The fine root turnover rate followed the order *T. ramosissima* > *A. sparsifolia* > *P. communis* > *R. soongorica* > *H. ammodendron* and ranged from 1.25 to 1.98 times/yr. The maximum fine root net productivity and fine root turnover rate were found for the *P. communis* and *T. ramosissima* communities (Table 3).

### 3.5. Environmental Factors Affecting SOC Dynamics

The relationships between SOC and ecological factors (soil water content, soil bulk density, soil conductivity, and pH) were significant (Table 4). According to the data, the SOC first decreased and then increased with increasing soil water content, soil bulk density, soil conductivity, and pH. When soil water content, bulk density, electrical conductivity, and pH were 21%, 1.36 g/cm^3^, 4.75 ms/cm, and 9.63, respectively, the SOC was lowest. 

Fine root biomass significantly affected the SOC values of all plant communities (Table 5). With increasing levels of fine root biomass, the SOC values first increased and then decreased; peak levels were reached at a fine root biomass of 12.5–45.0 g/m^2^. However, the change trend of SOC with fine root biomass in the *P. communis* community showed the opposite pattern, first decreasing and then increasing; at a fine root biomass of 277.77 g/m^2^, the lowest SOC value was reached.

## 4. Discussion

### 4.1. Relationship between the Vertical Distribution of and Seasonal Variations in SOC and Fine Root Biomass in Each Plant Community

The ability to predict and improve the consequences of global change partly depends on an improved understanding of the distribution and control of SOC, as well as how vegetation changes affect the distribution of SOC throughout the soil column [40]. Different vegetation types can significantly affect the vertical distribution of SOC [27,41,42] According to these scientists, SOC accumulation at deep soil layers in shrub-dominated environments is caused by vertical root dispersion. Based on global estimates, the SOC of desert ecosystems accounts for approximately 44% at a depth of 0–30 cm [41], and in past examinations in northwestern China, over half of the SOC content in shrub plots was aggregated at the 0–40 cm layer [14,43]. In the present study, the SOC levels ranged from 73–80% for the five plant communities and were therefore higher than the global estimate (Figure 5). As an explanation, different shrub species differ in their fine root biomass levels, leading to different SOC values [14,41,43]. For example, the fine root biomass in desert ecosystems accounts for approximately 61% of the 0–100 cm layer and is concentrated in the first 0–30 cm [41]. In the present study, the living fine root biomass was 64–86%, whereas the dead fine root biomass was 73–85%, above the global estimates (Figure 2). These results are similar to those reported by other authors, namely that over 60% of the fine root biomass is gathered in the initial 30 cm of soil [41,44,45,46]. In addition, in the present study, the live and dead fine root biomass at 40–90 cm accounted for 84.65% of the total live and dead fine root biomass for the *P. communis* community. This is in contrast to the results reported in a past study, where giant reed development occurred in the shallow soil layers; however, according to the authors, these herbaceous crops tend to extend an enormous extent of their fine root biomass to more profound soil layers [47]. In another study, giant reed development occurred in the deeper soil layers, related to the occurrence of groundwater [47]. In the present study, when fine roots were excavated, a large amount of groundwater was also found at greater soil depths.

In the current study, the highest SOC values of the *A. sparsifolia* (3.08 g/kg), *T. ramosissima* (2.35 g/kg), and *R. soongorica* (2.34 g/kg) communities were found in June, and the highest values of the *H. ammodendron* (2.25 and 2.31 g/kg, *p* > 0.05) community were found in June and September (Table 2). However, the lowest fine root biomass (43.17–329.89 g/m^2^) of the five communities occurred in June. Fine root biomass (66.29–523.41 g/m^2^) and living fine root biomass (48.45–405.75 g/m^2^) peaked in August and September in the five communities (Figure 3 and Figure 4). Due to the high temperature and low rainfall in summer, the growth of fine roots and the activity of soil animals and microorganisms were limited, with a consequent reduction in SOC mineralization. The SOC reached the maximum in June, when the fine root biomass was lowest. Generally, at the end of the growing season, microbial activity increases, which will increase SOC mineralization. Subsequently, the plants reduce nutrient input into the leaves and increase energy input into the roots, leading to a reduction in SOC. For another, fine root growth occurs mainly in July and August, consistent with the precipitation, which amounted to 30% of the rainfall for the whole year, resulting in a maximum fine root biomass in August and September. Thus, the peak of living fine root biomass reflects the responses of fine root production to rainfall. Some studies have shown that the majority of fine roots developed in spring and died off in summer because of the high temperatures [48], which may be a potential reason for the peak of fine root mortality in July and August. In addition, in the present study, although the fine roots of the *P. communis* community were concentrated in the 40–90 cm layer, the SOC was concentrated in the upper 0–30 cm, and the SOC content of the *P. communis* community was the lowest among the five communities. The species *P. communis* is a tall perennial grass species [49] and occurs in the form of standing litter in the late growing season, which reduces the input of litter, whereas litter and fine root decomposition mainly occur in the upper soil layer [42]. Generally, the deep soil layer has a large bulk density and a low porosity, reducing the activity of microorganisms and thereby impeding fine root decomposition, resulting in a reduction in the input of SOC.

### 4.2. Relationship between SOC and Ecological Factors

SOC stocks are influenced by a combination of environmental factors and organisms. In most cases, environmental factors, including soil temperature, available supplements, soil moisture content, soil texture, and soil pH, are closely related to fine root production and turnover [22,50,51]. The biological factors include fine root characteristics and microbial composition [41,52]. In their literature review, Gill and Jackson [22] reported that soil temperature is, to a great extent, connected with fine root turnover, but in point-scale studies, the effects of slight temperature differences in fine root production and turnover could be ignored, and temperature appears to affect the initiation of fine root production [53]. In this study, regression analysis showed that SOC was lowest when the soil moisture content, bulk density, conductivity, and pH were 21%, 1.36 g/cm^3^, 4.75 ms/cm, and 9.63 respectively (Table 4). Higher water content and soil bulk density are not conducive to aerobic respiration by aerobic microorganisms, resulting in the slower decomposition of organic matter [54,55,56]. Soil pH affects not only the dissolution of organic matter (fine roots), soil nutrients, and redox status (conductivity), but also the structure and activity of the microbial community. In general, at pH > 8.0, the activity of soil microorganisms decreases and root decomposition is slow [57,58,59]. In addition, the composition, structure, and physical condition of fine roots are closely related to SOC. In this study, fine root production, annual decomposition, and SOC replenishment followed the order *P. communis* > *H. ammodendron* > *A. sparsifolia* > *T. ramosissima* > *R. soongorica* (Table 3). In principle, simple organic substances such as monosaccharides, polysaccharides, and amino acids are easily converted to SOC by microorganisms, while complex organic substances such as lignin and polyphenols are difficult for microorganisms to use and do not contribute to the formation of SOC [60]. In summary, suitable soil moisture and high soil conductivity facilitate the decomposition of root litter and thus the formation of SOC [54,55]. Higher soil pH and soil bulk density levels are not conducive to soil biological activity and organic matter decomposition, and when they are more unfavorable to SOC mineralization, they lead to increased SOC levels [54,56].

### 4.3. SOC of Decomposed Fine Roots

In the present study, the fine root distribution patterns of the *A. sparsifolia*, *T. ramosissima*, *R. soongorica*, and *H. ammodendron* communities were similar to those in various arid areas (Figure 2, Figure 3 and Figure 4); generally, topsoil layers contain more fine roots [14,27,61,62]. In arid areas, a high fine root biomass in the top layer might increase the capacity of plants to retain soil assets (especially water) and adjust to dry season periods [62], whereas it might diminish their C investment [42]. In this study, there was a distinction in the fine root biomass among the different plant communities (Figure 2, Figure 3 and Figure 4), probably resulting from the hereditary differences [42]. Previous studies have demonstrated that communities with intense and shallow root systems and those with deep roots can coexist and interact with neighboring shrubs when resources are scarce [14]. In our study, the differences in the vertical distribution of fine roots among communities suggest that they might have different adaption mechanisms when resources are limited [6]. Fine root production varied among the different community plots (Table 3). Besides, the fine root production of *P. communis* was significantly higher than that of *A. sparsifolia*, *T. ramosissima*, *R. soongorica*, and *H. ammodendron*, most likely because in semi-arid and arid regions, the topsoil is at least generally or periodically dry [63]. Moreover, because of the heterogeneity of soil nutrients and water, fine root production shows different vertical patterns [64]. In sandy soils, the soil water content increases with depth, indicating that plants with a greater extent of fine roots at more profound depths can utilize water stored at in deeper soil layers, which explains the variation in parched regions [62,63].

An increase in SOC stocks fundamentally relies upon the rate of fine root decomposition [34]. The turnover rate in the present work ranged from 1.25 to 1.98 times/yr (Table 3), falling in the range of shrubs in temperate zones [8]. Huang obtained a fine root turnover rate (1.28–2.12 times/yr) similar to that observed here in the arid regions of northwestern China [27]. In a previous study, for temperate tree species across the eastern US, the average turnover rate was 1.27 times/yr, which was below the average turnover rate (1.6 times/yr) in the present study. Here, the turnover rate was closer to the fine root turnover rate (0.68–2.20 times/yr) of tropical forests. Generally, fine roots in arid regions have high turnover rates, which is an “adaptive strategy”. Fine root sloughing, with subsequent microbial immobilization, could help retain root nutrients and mineral elements in the upper soil horizons when plants experience drought, competition, and nutrient deficiency [65,66].

Our results show that a high content of fine root biomass and a high fine root production support the return of organic mass and nutrients through fine root turnover [67]. Nonetheless, higher fine root biomass and production were not related to a greater SOC content in communities (Figure 2, Figure 3, Figure 4, Figure 5 and Figure 6). Moreover, the SOC stocks did not respond to high fine root biomass [41], and soil C accumulation was not affected by the level of standing fine root biomass and its production [68,69]. A possible explanation is that in addition to fine root, leaf, and branch litter, the insoluble organic matter from roots, refractory microbial biomass, and living root exudates can, to a great extent, contribute to SOC accumulation at more profound soil depths in some regions [70,71] For example, some studies have indicated the importance of SOC originating from living roots as root exudates [71], and there is substantial evidence that living root inputs, not root or shoot litter, play a significant part in SOC formation [72]. Overall, these results suggest that the fine root litter of different communities contributes to SOC accumulation at different rates and that the mechanism of SOC accumulation varies among plant communities.

## 5. Conclusions

In this study, an asymptotic single-parameter global model was used to describe the depth distribution of the SOC reservoir (Figure 6), and the results implied that soil organic carbon levels were up to 70% below 0–30 cm depths in five plant communities. At the same time, the survey showed that live and dead fine root biomass at 0–30 cm accounted for 63.86–86.0% and 73.04–85.03% of the total fine root biomass (except for the *P. communis* community), respectively (Figure 2 and Figure 3). Regression analysis showed a significant positive correlation (*p* < 0.05) between the vertical distribution of SOC and fine root biomass (Table 5). Furthermore, SOC and fine root biomass showed a reciprocal relationship with seasonal changes, with SOC reaching a maximum (1.88–3.08 g/kg) in summer (June and July) (Table 2); however, the lowest values of fine root biomass were found in June (43.17–329.89 g/m^2^) (Figure 4). Subsequently, SOC declined to various degrees in August, September, and October (Table 2), while fine root biomass returned to its peak in August and September (66.29–523.41 g/m^2^) (Figure 4). The differences in fine root biomass and SOC among the different plant communities in this study were attributed to two factors. Regression analysis showed that soil water content and soil bulk density had a significant effect on SOC (*p* < 0.05) (Table 4). In addition, the results implied that the turnover rate of fine roots ranged from 1.25 to 1.98 times/yr (Table 3). Genetic differences in plant communities mainly resulted in fast and slow rates of fine root turnover and decomposition, as well as heterogeneity in soil texture and moisture, resulting in fine root production showing different vertical patterns and different mechanisms of soil carbon stock accumulation.

## Figures and Tables

**Table 1:** Characteristics of the five plant communities.

Dominant Species	Accompanying Species	Elevation (m)	Longitude & Latitude	pH
*Alhagi sparsifolia*	*Peganum harmala*	472	44°20.334′ N	8.65
	*Ceratocarpus arenarius*		88°00.308′ E	
*Tamarix* *ramosissima*	*Nitraria sibirica*	465	44°18.196′ N	9.29
	*Petrosimonia sibirica*		87°51.519′ E	
*Reaumuria soongorica*	*Ceratocarpus arenarius*	485	44°20.147′ N 88°07.807′ E	9.31
	*Salsolacollina*			
	*Haloxylon ammodendron*			
*Haloxylon ammodendron*	*Salsola collina*	462	44°19.094′ N	9.51
	*Nitraria sibirica*		87°50.329′ E	
*Phragmites communis*	*Salicornia europaea*	462	44°18.912′ N	8.73
	*Nitraria sibirica*		87°50.185′ E	

**Table 2:** Changes in the soil organic carbon (SOC) content over time (mean ± SD).

Month	Communities				
	*A. sparsifolia*	*T. chinensis*	*R. soongorica*	*H. ammodendron*	*P. communis*
June	3.08 ± 0.13 a	2.35 ± 0.16 b	2.34 ± 0.27 b	2.25 ± 0.31 b	1.19 ± 0.14 c
July	2.85 ± 0.28 a	2.15 ± 0.07 b	1.68 ± 0.18 b	1.62 ± 0.18 b	1.88 ± 0.17 b
August	2.79 ± 0.30 a	2.28 ± 0.06 b	1.82 ± 0.13 c	1.98 ± 0.15 bc	1.50 ± 0.23 c
September	2.81 ± 0.23 a	2.17 ± 0.12 b	2.21 ± 0.29 b	2.31 ± 0.28 ab	1.84 ± 0.13 c
October	2.94 ± 0.16 a	2.07 ± 0.26 b	1.72 ± 0.17 cb	1.99 ± 0.23 bc	1.35 ± 0.03 c

Note: soil organic carbon content (g/kg) (mean ± SD) of five communities at 0–100 cm. Each value represents the average of three replicates. Different lowercase letters indicate significant differences at *p* < 0.05 among the five plant communities.

**Table 3:** Fine root productivity, decomposition, decomposition of supplementary soil organic carbon (SOC), and turnover rate of the five plant communities.

Communities	Fine Root Production	Decomposition	Supplementary SOC	Turnover Rate
	(g/m^2^/a)	(g/m^2^/a)	(g/m^2^/a)	(Times/a)
*A. sparsifolia*	118.81 c	36.88 c	15.13 c	1.75 b
*T.* *ramosissima*	83.60 d	17.41 d	7.38 d	1.98 a
*R. soongorica*	50.67 e	16.08 e	7.14 d	1.41 d
*H. ammodendron*	168.02 b	57.29 b	25.08 b	1.25 e
*P. communis*	486.92 a	130.24 a	50.42 a	1.61 c

Note: different lowercase letters indicate significant differences at *p* < 0.05 among the five plant communities.

**Table 4:** Relationship between soil organic carbon (SOC) and ecological factors at 0–100 cm from June to October.

Ecological Factors	Regression Equation	*R* ^2^	Number of Samples
Soil water content (%)	y = 0.01x^2^ − 0.42x + 5.62	0.571 **	106
Soil bulk density (g/cm^3^)	y = 3.24x^2^ − 9.28x + 8.98	0.080 *	93
Soil electrical conductivity (ms/cm)	y = 0.06x^2^ − 0.57x + 3.77	0.026	67
pH	y = 0.88x^2^ − 16.95x + 83.91	0.084	67

Note: y, soil organic carbon (g/kg); x, ecological factors. Significance levels: **, *p* < 0.01; *, *p* < 0.05.

**Table 5:** Regression analysis of soil organic carbon (SOC) and fine root biomass at 0–100 cm from June to October.

Communities	Regression Equation	*R* ^2^	Number of Samples
*A. sparsifolia*	y = −0.002x^2^ + 0.18x + 1.29	0.582 **	30
*T. ramosissima*	y = −0.01x^2^ − 0.25x + 3.86	0.308 **	30
*R. soongorica*	y = −0.004x^2^ + 0.14x + 1.56	0.258 *	30
*H. ammodendron*	y = −0.001x^2^ + 0.06x + 1.12	0.204 *	30
*P. communis*	y = −0.000018x^2^ − 0.01x + 2.19	0.401 **	30

Note: y, soil organic carbon (SOC) (g/kg); x, fine root biomass (g/m^2^). Significance levels: **, *p* < 0.01; *, *p* < 0.05.

## Data Availability

Not applicable.
